# Embedded Data Imputation for Environmental Intelligent Sensing: A Case Study

**DOI:** 10.3390/s21237774

**Published:** 2021-11-23

**Authors:** Laura Erhan, Mario Di Mauro, Ashiq Anjum, Ovidiu Bagdasar, Wei Song, Antonio Liotta

**Affiliations:** 1 College of Science and Engineering, University of Derby, Derby DE22 1GB, UK; L.Erhan@derby.ac.uk (L.E.); O.Bagdasar@derby.ac.uk (O.B.); 2 Department of Information and Electrical Engineering and Applied Mathematics, University of Salerno, 84084 Fisciano, Italy; mdimauro@unisa.it; 3 College of Science and Engineering, University of Leicester, Leicester LE1 7RH, UK; A.Anjum@leicester.ac.uk; 4 Department of Computing, Mathematics and Electronics, “1 Decembrie 1918” University of Alba Iulia, 510009 Alba Iulia, Romania; 5 College of Information Technology, Shanghai Ocean University, Shanghai 200090, China; wsong@shou.edu.cn; 6 Faculty of Computer Science, Free University of Bozen-Bolzano, 39100 Bolzano, Italy

**Keywords:** Internet of Things, edge computing, data imputation, edge intelligence

## Abstract

Recent developments in cloud computing and the Internet of Things have enabled smart environments, in terms of both monitoring and actuation. Unfortunately, this often results in unsustainable cloud-based solutions, whereby, in the interest of simplicity, a wealth of raw (unprocessed) data are pushed from sensor nodes to the cloud. Herein, we advocate the use of machine learning at sensor nodes to perform essential data-cleaning operations, to avoid the transmission of corrupted (often unusable) data to the cloud. Starting from a public pollution dataset, we investigate how two machine learning techniques (kNN and missForest) may be embedded on Raspberry Pi to perform data imputation, without impacting the data collection process. Our experimental results demonstrate the accuracy and computational efficiency of edge-learning methods for filling in missing data values in corrupted data series. We find that kNN and missForest correctly impute up to 40% of randomly distributed missing values, with a density distribution of values that is indistinguishable from the benchmark. We also show a trade-off analysis for the case of bursty missing values, with recoverable blocks of up to 100 samples. Computation times are shorter than sampling periods, allowing for data imputation at the edge in a timely manner.

## 1. Introduction

Smart environments find themselves at the intersection of the Internet of Things (IoT) and cloud computing, and are capable of gathering information on the surroundings (monitoring), as well as manipulating it in order to accommodate certain conditions (actuation) [1]. Examples include, but are not limited to, smart cities, smart homes, smart grids, smart industry, smart health, and smart transportation. A challenge that arises is the management of the big IoT data generated by these types of systems [2]. It is receiving noteworthy attention across application domains, ranging from industrial IoT (IIoT) [3], to green energy [4], healthcare [5], Industry 4.0 [6], and many other domains [7]. Using only cloud-assisted computing may soon prove to be unsustainable, given the costs associated with storing, transmitting, and processing the sheer amount of raw (unprocessed) data produced by the sensor nodes. Therefore, we advocate for the use of solutions involving edge computing, a paradigm proposed for solving IoT and localized computation needs [8,9,10]. In this way, part of the processing can be done at the edge, close to the data source, which in turn results in costs savings related to data transmission, latency and bandwidth usage among other benefits. Examples of tasks in an edge computing scenario can include task-based resource allocation [11], service scheduling for power distribution [12], task offloading mechanisms [13], local sentiment analysis [14], charging, and discharging networking system algorithms for electric vehicles [15], etc.

Among the plethora of data management issues, the missing data imputation emerges as one of the most critical problems. The pertinent technical literature [16] proposes the following taxonomy of missing data management techniques: (i) missing data deletion; (ii) estimation of the missing data on the basis of modelling the known distribution (e.g., Gaussian Mixture Models, Expectation-Maximization); (iii) imputation or estimation of missing data through machine learning techniques.

In this work, we mainly focus on the environmental edge data imputation using machine learning approaches. Actually, applying machine learning techniques to the missing data problem is not new in the literature. For instance, in [17], the authors exploit artificial neural network models to impute data (in particular, anomalies) through time series data. Decision trees (DT) and CART algorithms are applied in [18,19], respectively, to solve the missing data issues. Other examples include support vector machines (SVM) and self organizing maps (SOM), which are exploited in [20,21], respectively. In all of the aforementioned works, the ML-based techniques are basically stressed to evaluate their accuracy, but the possibility of applying them directly on board the sensors has not been investigated.

Conversely, we propose the use of machine learning techniques at the sensor nodes, close to the data source, for cleaning incoming data by imputing missing values. This approach is motivated by the general goals of minimizing network and energy costs necessary for data transmission, as well as for increasing the reliability of the collected data.

As opposed to the classic approach of collecting the data from the sensors and then forwarding it to the cloud, we propose embedding pre-processing at the edge of the network. The growth of the IoT and the availability of computationally powerful devices, which can be deployed within the IoT environment, allow us to perform computationally intensive tasks at the edge, in order to increase data reliability and usability. In turn, this prevents the transmission of corrupted and unusable data further up the processing pipeline, including all the way up to the cloud. Additionally, further processing, depending on the application scenario, should be carried out at the edge after the data imputation process as to minimize the transmission of data across the network. Furthermore, additional checks can be performed at the edge to decide when and if the data should be transmitted to the next processing layer. One example for further processing at the edge, while making use of the edge imputed data, could be incorporating federated learning [22] on either the same edge device, or a neighbouring dedicated edge device. In this way, the new reliable data (as opposed to the raw, potentially contaminated and missing data) can serve as training data for the local training of different machine learning models.

As proof of concept for embedded data imputation at the edge, we investigate and evaluate the performance of two representative machine learning techniques, kNN and missForest, on the board of an IoT device, namely Raspberry Pi 4B (RPI 4B). We start with an artificially generated public pollution dataset, which serves as the benchmark. The dataset is representative of streaming data, as it is a sequence of data points generated by real-time data sources, which can be imperfect with missing data. The dataset is corrupted to different degrees as to account for two scenarios of missing data, namely random non-bursty missing data and random bursty missing data. kNN and missForest, two commonly used machine learning techniques, are benchmarked against two popular statistical based techniques, namely the mean imputation and multiple imputation by chained equation-MICE, for the task of filling in the missing data whilst taking into account the following metrics: root mean squared error (RMSE), density distribution, execution time, and RAM and CPU utilization. Our experimental evaluation is carried out on the board of a constrained environment, namely the RPI 4B, and on a laptop, to verify the computational trade-offs under limited computing conditions.

We find that kNN and missForest outperform the statistical based techniques and can correctly impute up to 40% of randomly distributed missing values, if we are to consider an RMSE of up to 10 as acceptable, given the environmental dataset we use. Furthermore, they are able to recover blocks of up to 100 missing samples in the bursty case scenario before their performance drops to that of mean imputation and MICE. Additionally, we provide a trade-off analysis for the bursty case, considering the chosen algorithm, dataset impairment rate and burst size. We also discuss time and space complexity for the different scenarios in terms of execution time and RAM and CPU utilization for the RPI 4B. The resulting execution times are shorter than the sampling period of the considered environmental IoT scenario, allowing us to show that data imputation can be achieved at the edge on board of constrained devices for our scenario. This encourages us to continue investigating how to take advantage of edge computing in order to optimize existing processing pipelines within the IoT, as well as to build on top of existing smart intelligent sensor systems.

In this work, we attempt to provide an answer to the question of “where” performing the data imputation techniques (e.g., closer to cloud or to devices). Such a question does not admit a unique response, since it strongly depends on the particular application and/or context. For instance, performing data imputation within the cloud could be attractive since we have virtually infinite computational resources; thus, we could neglect issues related to the time/space complexity of specific techniques. On the other hand, the cloud being “far” from the devices, some local correlations among measurements could be lost, and the data imputation could be inaccurate. The aforementioned benefits and drawbacks are reversed if we decide to perform data imputation techniques directly on board the devices. In our analysis, we try to quantify such differences through an experimental comparison with data imputation techniques applied close to cloud (e.g., on a fixed and powerful platform) and close to the edge (namely on board the Raspberry PI).

The paper is organized as follows: Section 2 places our work within the literature. Section 3 mainly focuses on the methodology and the chosen dataset, the scenarios designed for impairing the dataset, the techniques adopted for the edge data imputation, as well as the cases considered for the experimental work. Section 4 presents the results and findings for the considered scenarios, while Section 5 discusses important considerations as part of the undertaken experimental work. Section 6 concludes the present paper and outlines directions for future work.

## 2. Related Work

The missing data imputation problem in IoT has received significant attention in recent years. Precisely, two main tracks of research emerge: the first one is focused on a cooperative approach, where one attempts to identify space/time correlations among data acquired by the IoT devices, so that measurements missing from a sensor can be replaced by measurements from other correlated sensors. The second one focuses on specific techniques/algorithms, which typically borrow crucial concepts from statistics and/or machine learning.

In line with the former track, the work in [23] advances a double layered clustered scheme along with a consensus-based framework aimed at substituting missing values from the sensors measurements. In particular, the nodes located at the edge perform the data imputation. Such an imputation is evaluated by considering the correlation between the measurements affected by missing values coming from a given sensor (e.g., sensor *A*), and the “healthy” measurements collected by other sensors located in the proximity of sensor *A*.

A similar approach, relying on the spatiotemporal correlations, has been adopted in [24], where a comparison among several data imputation techniques has been proposed. Yet, the authors in [25] propose a data imputation strategy that relies on the concept of “group opinion”. For instance, metrics, such as the Mahalanobis distance and the cosine similarity, are combined to evaluate the data replacement proposed by a group of peer devices. Finally, the group and the local opinions are aggregated through a weighting mechanism to propose the best replacement.

A similar concept based on the opinion of a group of devices is exploited in [26], where a continuous correlation detection methodology is applied in real-time to streams of data coming from IoT devices. The temporal–spatial correlation, jointly with a kNN algorithm, is exploited in [27], where the spatial correlation of sensor data are described through a linear regression model, and where information from multiple neighbouring nodes is used to estimate the missing data jointly, rather than independently.

Guastella et al. propose in [28] an approach based on multi-agent system (MAS), which allows distributing the computation among the local agents grouped in regions for imputing missing environmental data during the data collection process for large-scale systems. The imputation is based on the two-dimensional inverse distance weighting (IDW) interpolation for irregularly spaced data. The obtained values are compared to those from other agents in the same Voronoi region to determine if they are correct or anomalous. The validity of the Voronoi partitioning is tested with every iteration.

In line with the second research track is the work in [29], where the incomplete sensed data across the IoT world is managed through the probabilistic matrix factorization (PMF) method, along with the usage of a K-means algorithm to measure the similarity among neighbouring sensors.

In [30], the authors combine the expectation maximization (EM) algorithm and data augmentation operations to perform multiple data imputation for missing values in a land price dataset.

In [31], the missing values imputation within sensor-based measurements is performed through the Bayesian maximum entropy (BME) technique. The performance of the BME technique seems to outperform the PMF in terms of accuracy, time efficiency, and robustness.

The authors in [32] face the missing data problem in IoT systems by focusing on the common mode failure problem, meaning that a single event can lead to the loss of data from numerous sensors at the same time. Accordingly, they advance a specific technique to deal with the large gaps in univariate time-series data, along with an iterative framework nicknamed *Itr-MS-STLecImp*, which acts according to the two steps: gap segmentation and gap reconstruction.

A Gaussian mixture model (GMM), to handle missing values in IoT systems, has been advanced in [33]. In particular, the authors proposed the recovery of 21 missing temperature sensor values from a set of 220 observations.

A sophisticated neural-based approach is used in [34], and relies on a combination between the general regression neural networks (GRNNs) and the successive geometric transformation models (SGTMs) to solve the problem of completing missing data from IoT devices.

Authors in [35] specifically tackle the problem of data reconstruction across wireless sensor networks, where data loss is commonly due to noise, unreliable links, or collisions. To address such problems, they propose an algorithm dubbed *ESTICS*, which exploits advanced concepts of the compressive sensing theory to reconstruct the massive missing data.

Finally, the singular spectrum analysis (SSA) to maximize the accuracy of data imputation in IoT-based surveillance environments is advanced in [36], where a non-parametric spectral estimation along with spatial–temporal correlations of time-series data from IoT devices are exploited together.

While all the works discussed earlier present interesting and innovative techniques or frameworks to manage the problem of missing data, some important differences arise with respect to our proposal. First, in many works (see, e.g., [25,26,27,28,30,31,32,33,35,36]), despite the data coming from the IoT, the analysed imputation algorithms actually run on standard computer architectures (e.g., PCs, laptops, etc.). Moreover, the importance of tackling the missing data problem as close as possible to the devices is evident (e.g., at the Edge of the network [37]); thus, our assessments are carried out straight on the board of the devices. Second, many existing experiments (see, e.g., [24,25,26,32]) are performed by simplistic data missing models, while we consider the problem of bursty missing values, which often arises when a sensor becomes unavailable for a certain (finite) period of time (a common situation for environmental sensors). Finally, compared to other works (see, e.g., [23,24,34,35]), which only account for one performance analysis (for instance, the imputation method accuracy), in our approach, we complement this analysis with a time assessment, which seems critical within real-time environmental data. Table 1 summarizes the main elements of novelty of our proposal with respect to some existing literature.

## 3. Methodology

In this section, we explain the environmental dataset (Section 3.1), the data corruption scenarios (Section 3.2), the techniques chosen for performing the data imputation (Section 3.3), and the experiments design (Section 3.4).

### 3.1. Dataset Choice and Description

To analyse and carry out the experiments for edge data imputation, we chose an artificially generated IoT dataset, namely *Pollution Measurements (generated data)*, part of the *City Pulse Smart City Datasets* [38]. The dataset was created so as to complement the traffic level data (real dataset of vehicle traffic observed between two points for a set duration of time) in the city of Aarhus, Denmark. It is representative of streaming data, as it is a sequence of data points, with associated timestamps, generated by real-time data sources, which can be imperfect with missing data. Additionally, the data can suffer changes over time, as the monitored environment changes.

The synthesized pollution data consists of observations for the air quality index, which simulate a pollution sensor attached to each traffic sensor in the traffic dataset. The values are generated every 5 min using a pre-selected pattern http://iot.ee.surrey.ac.uk:8080/datasets/pollution/readme.txt, accessed on 10 February 2021: each sensor measurement (e.g., ozone level) is initially assigned a value between 25 and 100; every 5 min, the values will be updated as follows: if the previous value was outside the 20 to 210 range, then a random integer between 1 and 10 is added or subtracted, as to keep the new value in the desired interval or as close as possible to it; for all other cases, a random integer between −5 and 5 is added to the previous value.

**Figure 1:**
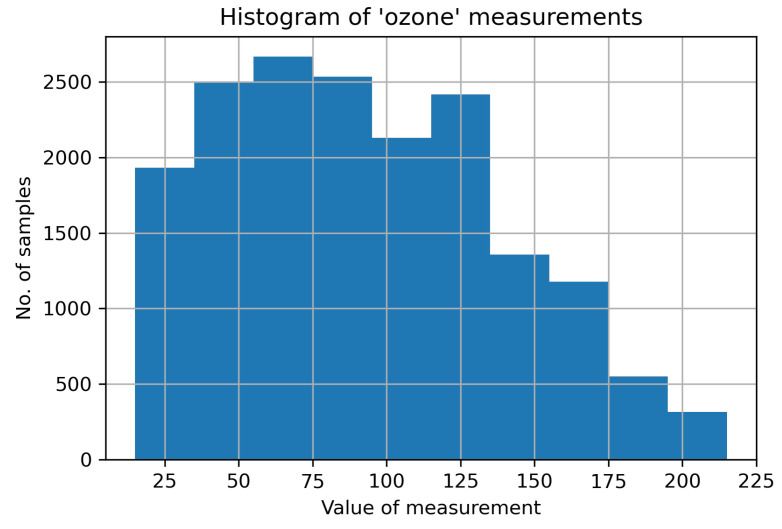
Histogram of the ozone measurements in the dataset.

For this work, from the aforementioned dataset, we chose the data corresponding to only one sensor. The dataset comprises 17,568 samples. Each sample has a set of environmental measurements (e.g., ozone level, carbon monoxide, etc.) along with location data (longitude and latitude), and timestamp. For the simplicity of the analysis, we dropped the location data from the dataset, as well as the timestamp (the order of the samples assures the time continuity of the samples), and retained only the pollution measurements. A description of the dataset is given in Table 2, where "std" stands for standard deviation, whereas 25%, 50%, 75% represent the first quartile, the second quartile (median), and the third quartile, respectively. For simplicity, we impaired only the ozone values and, thus, applied data imputation to the time series only. Figure 1 depicts the histogram of the original (unimpaired) ozone measurements.

### 3.2. Dataset Impairment

In its original form, the dataset used in this work can be considered curated and with no missing values. To test and compare different methods for data imputation at the edge, we impaired the dataset by introducing missing data in two different ways, which mimic two scenarios, as described in Table 3: the *non-bursty* scenario and the *bursty* scenario. On one hand, for the non-bursty case, individual data points are randomly chosen to be invalidated within the dataset. For the bursty case, a corresponding number of bursts of a given size of data points are randomly chosen to be invalidated. The impairment level is expressed in terms of corrupted data points. In Table 3, we also provide a visual example for the way in which the data points are invalidated in the two cases. These type of errors are often encountered in an IoT setting.

### 3.3. Methods Chosen for the Edge Data Imputation

In this work, we analyse and compare well-known and readily available techniques in order to showcase the simplicity and ease of deployment as part of pushing the data processing to the edge. We chose two machine learning based techniques (kNN and missForest), evaluating them against two statistical based methods (mean and MICE). In this way, we want to highlight that machine learning based techniques can achieve a good performance level in an edge environment without special modifications.

**Mean imputation** is perhaps one of the most common and straightforward approaches, where missing values are replaced with the mean of the considered variable. In our case, the missing data for the ozone measurements is replaced with the mean of the observed ozone values. However, one must be aware that, often enough, this method is not producing good enough results, as it changes the standard deviation, and it does not account for the relationship among the variables.

**Multiple imputation by chained equations (MICE) data imputation** is a robust, statistical, principled, multiple imputation technique. It works by making multiple predictions for each missing value. The procedure fills in the missing data through an iterative series of predictive models, as explained in [39]. Azur et al. provide a comprehensive analysis and description of the chained equation approach to multiple imputation in [40], as well as an overview of the steps the MICE algorithm undertakes for convergence. In this work, we use the python library function *impyute.imputation.cs.mice* that differs from the implementation proposed by Buuren et al. in [41] in two aspects, namely stopping criterion and variable to regress on https://impyute.readthedocs.io/en/latest/_modules/impyute/imputation/cs/mice.html, accessed on 25 March 2021. We apply the technique on the whole dataset (consisting of the five columns, as described in Table 2).

**missForest data imputation** is an iterative imputation method, based on a random forest, and has been introduced in [42]. It works by averaging over a number of different decision trees (unpruned classification or regression trees). In this work, we use the *missForest* method, part of the *missingpy* Python library. We apply the technique on the whole dataset (consisting of the five columns, as described in Table 2).

**kNN data imputation** works by filling in missing data points based on the values of its closest *k* neighbours, identified through the usage of the euclidean distance [43]. In this work, we use the *KNNImputer* method, part of the *sklearn.impute* Python library. We apply the technique on the whole dataset (comprising the five columns, as described in Table 2). We chose a *k* value of 3 in order to keep the search of neighbours to a minimum. This can be further optimized by analysing the impact of the *k* value over the performance in relation to the time and space complexity.

### 3.4. Experiment Design

The experiments used to evaluate and compare the performance of the chosen techniques for the data imputation correspond to the impairment methods described in Table 3.

For the **non-bursty case,** we compare the performance of the data imputation methods in the context of an impairment rate varying from 1% to 99%. A step of 5% is used from the impairment rate of 5% until that of 95%. From the rate of 95% until that of 99%, a step of 1% is used.

For the **bursty case,** the methods consider a burst size varying from 5 to 200 with a step of 5. We also include the non-bursty case scenario with burst size 1 for comparison. The impairment rate is kept within the 1% to 25% range.

An important aspect of our experiment design is given by the environment used. In order to showcase the possible use of these data imputation techniques at the edge, within the IoT ecosystem, we make use of a Raspberry Pi 4B with 4 GB of RAM [44]. The RPI 4B is used to run the experiments and collect metrics for the execution time and memory usage. The experiments are also carried out on a laptop for comparison purposes. The technical specifications for the laptop and the Raspberry Pi 4B are highlighted in Table 4. Part of the experiments, which do not concern measuring execution times, are run exclusively on the laptop. Moreover, all graphics presented in this work were generated on the laptop.

The setup of the RPI 4B for this work included installing the *Raspbian GNU/Linux 10 (buster)* operating system on the device. The code for the experiments was written and executed both on the laptop and the Raspberry Pi with *Python3.7*.

## 4. Result Analysis

In this section, we present and discuss the results of the experiments. We group the results based on the two scenarios: non-bursty case and bursty case. We compare the two machine learning based techniques with the statistical based methods for the task of data imputation by considering the RMSE, execution times, and the data distribution. Furthermore, we discuss the CPU and RAM usage for the RPI 4B.

### 4.1. Non-Bursty Case

**Figure 2:**
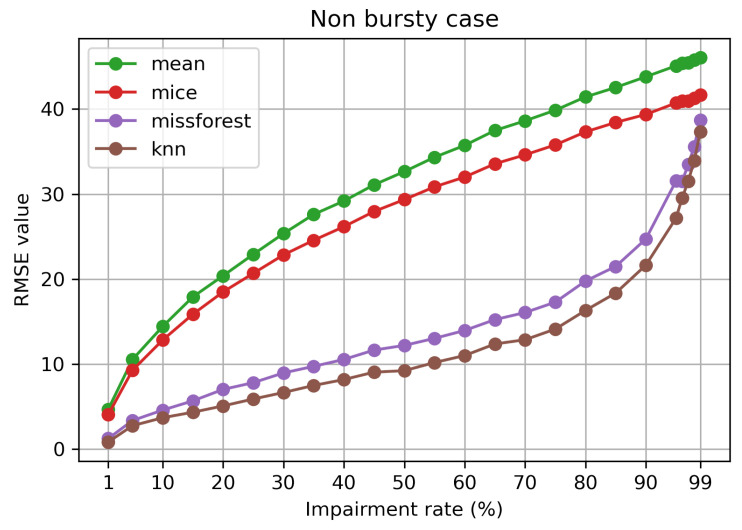
RMSE value in relation to impairment rate (%) for the non-bursty case.

Figure 2 depicts the evolution of the RMSE in relation to the impairment rate, which varies from 1% to 99%. As expected, the mean imputation performs the worst, as it does not consider anything else except the mean of the dataset. MICE imputation performs slightly better in comparison to mean. However, both techniques experience a rapid and steady growth with the increasing amount of missing data. On the other hand, missForest and kNN experience the worsening of performance with the increase of the impairment level at a much slower rate. Only after an impairment rate of 75%, there is a more rapid increase, with the two methods eventually catching up to the other two. Additionally, considering a desirable RMSE of at most 10, mean and MICE imputation can handle an impairment level of at most 5%; however, missForest and kNN can handle missing data of up to 40 and 50%, respectively. We discuss of a RMSE value of at most 10 given the environmental dataset we use, and in order to outline the differences between the four techniques.

**Figure 3:**
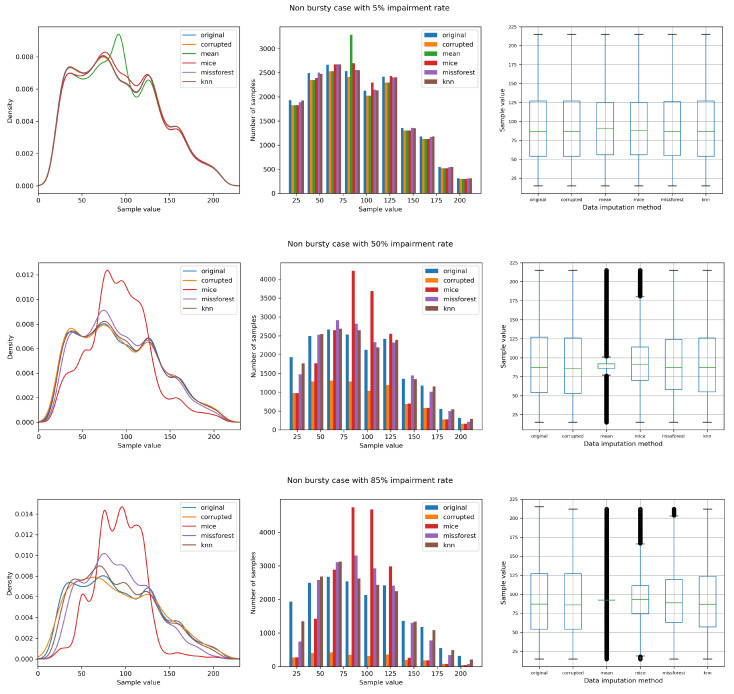
Non-bursty case comparison with 5%, 50%, and 85% impairment rate.

Figure 3 shows the data distribution by means of density plots, histograms, and box plots of the datasets accounting for the original, contaminated, and after the imputation state, for 3 different impairment levels. Starting from the top (5% of missing data), most techniques stay close to the original dataset. However, one can already notice the disadvantages of the mean imputation (the increase of distribution around the mean value). From the other plots, the line representing the mean imputed dataset is not included, to avoid skewing the plot. On the other hand, the behaviour of the mean imputation method is shown in the box plots, where the poor performance emerges especially for growing impairment rates.

For an impairment rate of 50%, it can be noticed that missForest and kNN remain fairly close to the original dataset, with MICE not being able to recover missing data as accurately. When the corruption level reaches 85%, which can be considered an extreme condition, both missForest and kNN experience a significant drop in performance, particularly towards the interval ends; nevertheless, they recover missing data better than mean and MICE.

### 4.2. Bursty Case

**Figure 4:**
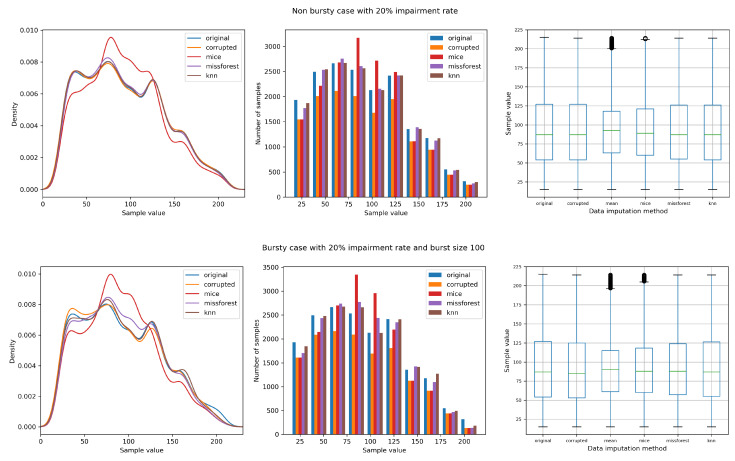
Non-bursty case and bursty case for the same impairment rate (20%).

Figure 4 allows us to compare the data distributions for the same impairment rate between the non-bursty case (burst size of 1) and the bursty case with a burst size of 100. It is observed that data that are missing in chunks have a greater impact on the performance of the data imputation methods, particularly on the machine learning based methods, as it can be noticed in the density plot.

**Figure 5:**
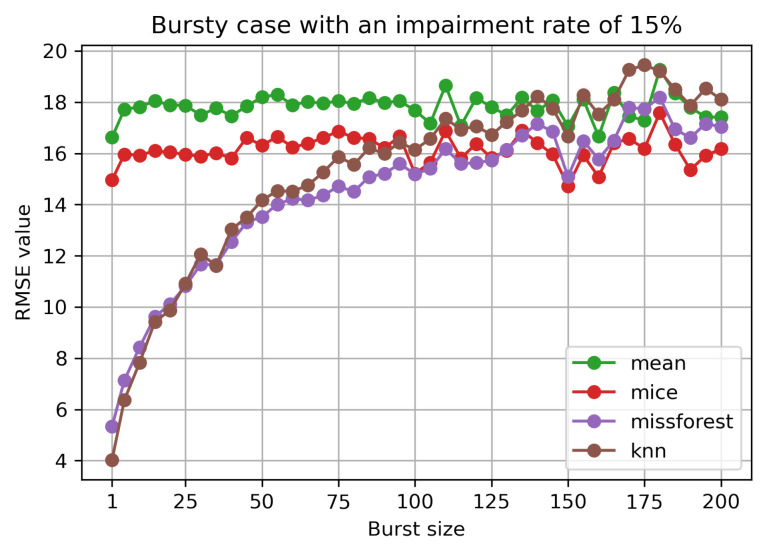
RMSE value for bursty case with 15% impairment rate.

In Figure 5, we observe the evolution of the RMSE for a 15% set impairment level, but varying burst size. The mean and MICE methods are not sensitive to the bursty case scenario, as opposed to missForest and kNN. The machine learning based methods have similar performance. For the two, a decrease in performance can be noticed with the increase of the burst size. They only catch with the other two methods for a burst size of around 100. The same behaviour is noticed for different impairment rates.

**Figure 6:**
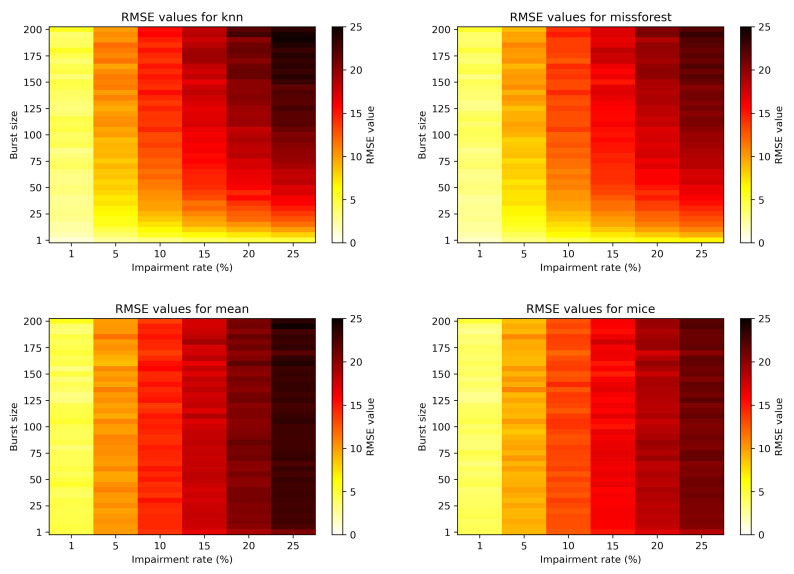
Colormap showcasing the RMSE in relation to the impairment rate and burst size.

Figure 6 depicts the more general view of the RMSE evolution with burst size and impairment rate for the four algorithms. Again, it is obvious that the burst size does not affect statistical based methods, but impacts the machine learning based methods. Moreover, for the bursty case scenario, the missForest imputation performs slightly better than kNN.

### 4.3. Time and Space Complexity

**Figure 7:**
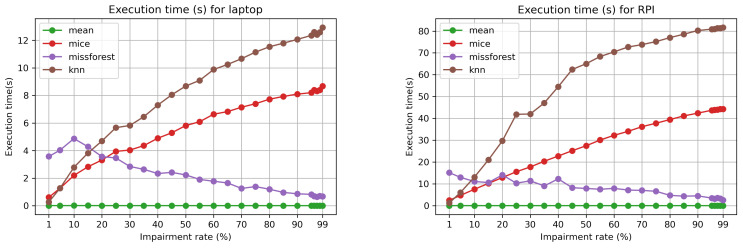
Execution times (s) on laptop and RPI 4B 4GB for the non-bursty case and varying impairment rates.

For the non-bursty case, we showcase and compare the execution time for each impairment rate while considering, as the running environment, both the laptop and the RPI 4B. This is depicted in Figure 7. Mean imputation has a constant time of below 0.01 s for the laptop, and of approximately 0.02 s for the RPI 4B. For both kNN and MICE imputation, the execution time goes up with the increase of the impairment rate. However, for missForest, the execution time decreases due to the complexity drop of the dataset (higher degree of missing data), which results in less computationally intensive tasks for building the necessary decision trees. The maximum execution time for the RPI in the non-bursty case is around 80 s, below the sampling time window of 5 min. Hence, this would allow for near real-time data imputation within the IoT scenario of our chosen scenario and pollution dataset.

**Figure 8:**
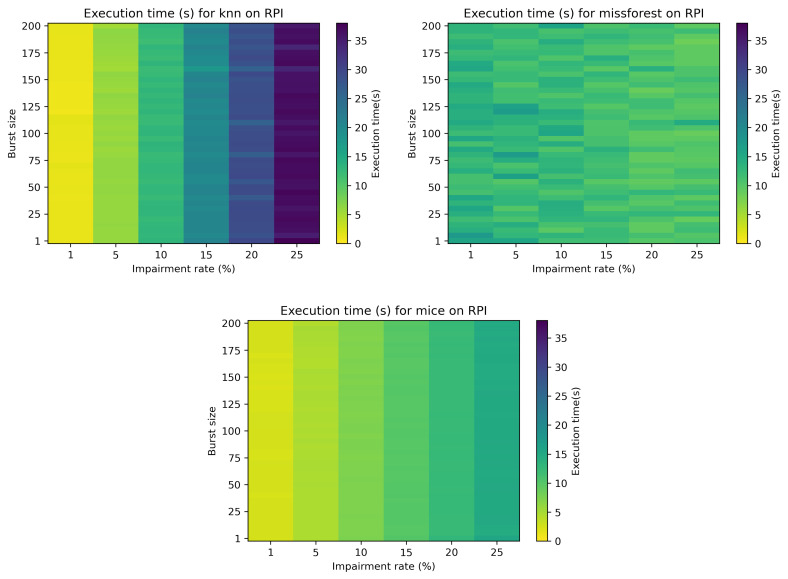
Colormap showcasing the execution time (s) in relation to the impairment rate and burst size for kNN, missForest and MICE data impuation.

Figure 8 depicts the execution times for the RPI for the bursty case, considering both the impairment rate and the burst size. The execution time for mean imputation is not depicted, as it is constant, as per the non-bursty case (approximately 0.02 s). It can be noticed that for kNN and MICE the execution times are not affected by the burst size, while missForest is only slightly affected in some cases.

**Figure 9:**
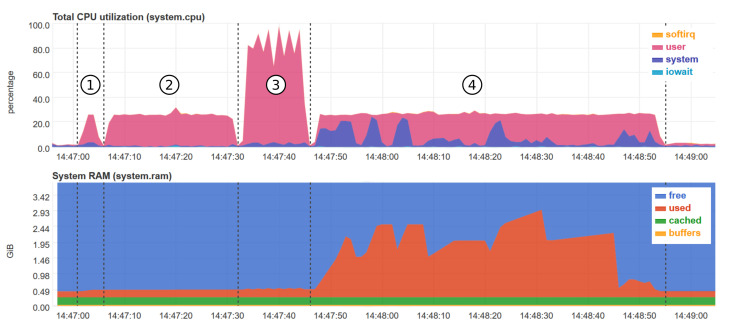
Snapshot of the CPU and RAM memory usage for the non-bursty case with 50% impairment rate on the RPI 4B (4GB of RAM) for each algorithm (1-mean imputation, 2-MICE imputation, 3-missForest imputation, 4-kNN imputation).

Figure 9 provides a snapshot of the CPU and RAM usage of the RPI for the non-bursty case with 50% impairment rate. The snapshot was taken by making use of the *netdata* platform, a tool for real-time monitoring https://www.netdata.cloud/, accessed on 25 March 21. missForest is the only method that takes advantage of the four cores of the RPI 4B. The other techniques use only one core at maximum, amounting to roughly 25% of the total CPU power. In terms of RAM usage, kNN imputation uses up to 3 GB of RAM for 50% impairment rate. kNN imputation can be carried out up to 30% impairment level also on a RPI 4B with 2 GB of RAM. The other techniques have a fairly constant RAM usage of maximum 0.5 GB.

## 5. Discussion

The performed experiments led us to a number of insightful observations. With regard to performance, kNN and missForest clearly outperform the remaining algorithms, due to their particular structure. For instance, it is useful to recall that both kNN and missForest are non-parametric techniques; thus, they perform well when there is no particular assumption about the data to impute. Conversely, MICE assumes that the data generating process originates from a parametric distribution. In particular, it assumes a linear relationship among the involved variables (rarely met in real-world cases), which causes an accuracy decay. Finally, the mean imputation method is the worst in terms of accuracy, mainly due to the fact that such a technique typically causes a strong variance alteration of the mean-imputed variables.

Regarding the time complexity analysis, a known weakness of the kNN is the computational time, mainly due to the fact that the data need to be calculated and sorted, leading to a complexity order of O(*n* log *n*). Although MICE is (slightly) faster than kNN, it relies on an imputation process repeated until all the missing data are estimated, resulting in a non-negligible computation time. In contrast, missForest can benefit from a strong parallelization, whereas mean imputation takes advantage from a very simple implementation, resulting in very competitive execution times.

Considering the non-bursty case scenario, and by solely looking at the RMSE, one would designate kNN as the winner method. Taking into account the bursty case, one notices that the differences between kNN and missForest are minimized, with missForest even slightly taking over, in terms of performance. At this point, we could say that actually both techniques are a winner, providing good performance compared to the more classic approach of mean and MICE. However, the situation significantly changes when we take into account both execution times and RAM usage, factors that are of great importance when considering edge computing and near real-time performance. Moreover, missForest appears to better suit the limited resources of an edge device, such as the RPI 4B for impairment rates larger than 10%, as it does not require large amounts of RAM and has a faster execution time. However, for lower impairment rates, the execution times for kNN imputation are lower than the ones for missForest and the RAM usage is not as heavy.

Another important point for deciding the best algorithm is the possible optimizations that could minimize resource consumption and decrease execution time. All techniques presented in this paper were used without additional optimizations, in order to offer a ground truth and bare-bones evaluation. A key optimization method would be to perform data imputation on time windows, as opposed to the whole dataset. This would in turn reduce the computation required, as well as the use of computational resources. Furthermore, the accuracy and performance of the techniques should not be negatively impacted in scenarios similar to our dataset (i.e., IoT pollution measurements data), if the time window is chosen to account for enough neighbouring data and any necessary existing patterns. Time windows could also allow for a parallelization of the other algorithms (mean, MICE, kNN), which in turn would result in smaller execution times.

## 6. Conclusions and Future Work

In this paper, we tackled the task of dealing with missing data within IoT smart environments at the edge for a set scenario. We proposed taking advantage of the edge in order to perform essential data cleaning, in terms of data imputation, as to increase reliability and usability of data. We investigated and evaluated kNN and missForest, two machine learning based techniques, on imputing missing data from an environmental IoT setting within two scenarios (bursty and non-bursty missing data). The two are benchmarked against two statistical based methods, namely mean imputation and MICE, considering the following metrics: RMSE, density distribution, execution time, RAM, and CPU utilization. The experimental work was carried out close to the data source (sensor nodes), on board of a constrained IoT device, namely the RPI 4B. kNN and missForest outperformed the other two techniques, being able to cope with impairment rates of up to 40% for the non-bursty case, as well as being able to recover blocks of up to 100 missing data samples for the bursty case before dropping to the performance level of mean and MICE.

Execution times were shorter than the sampling period of the considered environmental IoT scenario, allowing us to show that near real-time data imputation can be achieved at the edge, on board of constrained devices. This encourages us to continue exploring how to take advantage of edge computing, for optimizing existing processing pipelines in the IoT, as well as to build on top of existing smart intelligent sensor systems.

Future work can be pursued along the lines of optimizing the techniques for the conditions of the environmental scenario at hand, as well as to make use of time windows for the data imputation step, such that we could minimize execution needs. In our work, all chosen data imputation methods have not been optimized, or tuned in any way, in order to offer a ground truth and bare-bones evaluation. However, we believe that an analysis considering different time windows could help to further bring down the execution time, as well as lead to further increases in accuracy.

Moreover, it would be worth investigating the performance of the edge data imputation methods in the scenario of data missing from multiple data columns (multivariate imputation). In our work, for simplicity, we considered the case where the environmental collected data had missing values only in the ozone dimension. However, in real situations, data would be missing from any of the sensors of the systems. We expect that in this situation, further optimization can be done, so as to take advantage of the possible correlation between different neighbouring sensors. More importantly, we believe that future work should focus on different methods and scenarios in which one can optimize existing processing pipelines by pushing part of the data processing at the edge of the network, as there are many benefits, such as important savings on energy, storage space, and transmission costs.

Furthermore, an interesting avenue for expanding this work would be looking at different types of data collected by sensors, such as health data. The use of different datasets would allow for the expansion and evaluation of the suggested approach to other application scenarios. Moreover, besides different datasets, different computationally powerful IoT devices could be evaluated along the Raspberry Pi 4B, as other application domains could already have promising IoT devices as part of their overall infrastructure.

Finally, a comprehensive comparative evaluation of the most promising machine learning based techniques from past years, for the task of edge data imputation, would prove to be valuable for the research field. Our work could benefit from comparing our chosen algorithms against a multitude of other techniques to discuss advantages and disadvantages, and make recommendations for the appropriate techniques to use, depending on the application scenarios and technical requirements.

## Figures and Tables

**Table 1:** Summary of differences/limitations of surveyed work with respect to our proposal.

	Main Limitation	Our Approach
[25,26,27,28,30,31,32,33,35,36]	Data imputation techniques are applied within the IoT realm, where the experiments run on fixed platforms (sometimes through expensive tools such as Matlab or SPSS).	Imputation algorithms run directly onboard of sensors which are the main source of data.
[24,25,26,32]	Classic missing error models are used, with the problem of bursty missing values not addressed.	The problem of bursty missing values is explicitly faced since it represents a real-world scenario in which a sensor could be unavailable for a certain period of time.
[23,24,34,35]	The data imputation techniques are evaluated through performance indices (e.g., accuracy), but a time assessment is missing.	Performance analysis is complemented with a time assessment.

**Table 2:** Description of the dataset pollution measurements.

	Ozone	Particulate Matter	Carbon Monoxide	Sulphur Dioxide	Nitrogen Dioxide
count	**17568**	17,568	17,568	17,568	17,568
mean	**92.42**	106.12	100.54	131.66	159.18
std	**46.18**	52.01	49.66	50.51	43.43
min	**15**	15	15	15	18
25%	**54**	60	56	99	134
50%	**87**	107	99	131	173
75%	**127**	146	138	177	193
max	**215**	215	215	215	215

**Table 3:** The methods used for corrupting the dataset.

Type of Introduced Error (Random)	Description
**Non-bursty case**	We randomly select the individual data points to be invalidated from the dataset, in order to reach the desired dataset impairment level.
	● ● ● ● ● ● ● ● ● ● ● ● ● ● ● ● ● ● ● ●
	20 data points, desired impairment rate of 20% (4 points to be invalidated)
	●- original data point, ●- invalidated data point (N/A)
**Bursty case**	We randomly select a corresponding number of bursts of a given size (number of data points) to be invalidated from the dataset, in order to reach the desired dataset impairment level.
	● ● ● ● ● ● ● ● ● ● ● ● ● ● ● ● ● ● ● ●
	20 data points, desired impairment rate of 30% with burst size = 3 (6 points to be invalidated as part of 2 bursts of size = 3 data points)
	●- original data point, ●- invalidated data point (N/A)

**Table 4:** Hardware specifications for the experimental environment.

Hardware Specifications	RPI 4B	Laptop
**RAM**	4 GB	16 GB
**CPU**	Broadcom BCM2711, quad-core Cortex-A72 (ARM v8) 64-bit SoC @ 1.5 GHz	Intel(R) Core(TM) i7-8850H CPU @ 2.60 GHz

## Data Availability

Publicly available datasets were analysed in this study. This data can be found here: http://iot.ee.surrey.ac.uk:8080/datasets.html#pollution (accessed on 19 November 2021).
